# Dysphagia as a Postoperative Complication of Anterior Cervical Discectomy and Fusion

**DOI:** 10.7759/cureus.26888

**Published:** 2022-07-15

**Authors:** Georgios Tsalimas, Dimitrios Stergios Evangelopoulos, Ioannis S Benetos, Spiros Pneumaticos

**Affiliations:** 1 5th Orthopaedic Department, KAT General Hospital of Athens, National and Kapodistrian University of Athens School of Medicine, Athens, GRC; 2 3rd Orthopaedic Department, KAT General Hospital of Athens, National and Kapodistrian University of Athens School of Medicine, Athens, GRC; 3 Orthopaedics, KAT Trauma Hospital, University of Athens, Athens, GRC; 4 Orthopaedics, KAT General Hospital Of Athens, National and Kapodistrian University of Athens School of Medicine, Athens, GRC

**Keywords:** acdf complications, postoperative dysphagia, acdf, anterior cervical discectomy and fusion, dysphagia

## Abstract

Anterior cervical discectomy and fusion (ACDF), despite its possible complications, remains the gold standard for the surgical treatment of patients with radiculopathy and/or myelopathy caused by cervical intervertebral disc herniation or spondylosis. Despite its high rate of incidence, postoperative dysphagia following ACDF is still poorly understood; its pathogenesis remains relatively unknown, and its risk factors are still a subject of debate.

The aim of this study is to review the incidence, pathogenesis, diagnosis, and methods of prevention of dysphagia in ACDF patients. To this end, a literature review was conducted based on the PubMed internet database. Article titles were searched by using the following keywords: “dysphagia” and “anterior cervical discectomy and fusion” or “ACDF”. The search was limited to prospective clinical studies evaluating dysphagia after ACDF surgery. Studies published in non-English languages, retrospective studies, cadaveric studies, reviews, case reports, study protocols, and commentary studies were excluded.

Initially, 335 studies were identified after a primary search. After the application of the exclusion criteria, 73 studies remained for the final analysis. This literature review focused on identifying the rate of dysphagia and the various risk factors leading to this complication by comparing and evaluating the current literature with a wide spectrum of heterogeneity concerning patients, surgeons, and surgical techniques. A mean dysphagia rate of 19.4% (95% CI: 9.6%-29.1%) based on the findings of the included studies correlating dysphagia directly with ACDF procedures was calculated. Various established risk factors leading to dysphagia include the female sex, smoking, the surgical approach, rhBMP-2 use, and multilevel surgery, while zero-profile devices seem to reduce dysphagia risk. The diagnosis is based on clinical and radiological findings, especially prevertebral soft-tissue swelling. However, videofluoroscopic and endoscopic studies have been recently used for the evaluation of dysphagia. The role of local administration of steroids in the prevention of dysphagia has not yet been clarified. This review underscores the prevailing rudimentary understanding of the problem of dysphagia after ACDF procedures and highlights the need for more sensitive, factor-specific studies for understanding the impact of various risk factors on the incidence rate of dysphagia.

## Introduction and background

In spite of the potential complications associated with it, anterior cervical discectomy and fusion (ACDF) currently remains the gold standard in the surgical treatment of patients with radiculopathy and/or myelopathy caused by cervical intervertebral disc herniation or cervical spondylosis. ACDF maintains the disc height and the cervical alignment and the biomechanics of the cervical spine, besides promoting vertebral ossification and the faster return of the patients to daily activities. It is highly indicative when fixed cervical kyphosis >10 degrees is present, the compression arises from two or fewer disc segments, and anterior compression pathology is established and evident [ossification of posterior longitudinal ligament (OPLL), soft discs, disc osteophyte complexes]. Despite the fact that a large number of ACDFs are performed annually worldwide, various issues regarding the most appropriate intraoperative and postoperative management of these patients remain controversial and insufficiently defined. These include the method of fusion, the type of implants, the placement of plates and screws, the use of external vertebral orthotics, the application of postoperative physiotherapy, as well as the most appropriate duration of patients’ absence from work. Since there are no official, evidence-based guidelines, spine surgeons are often forced to follow their own tactics, based on their training, specialization, and clinical experience [1-3].

The anterior cervical approach is a technically safe method, in which the structures in the anterior and anterolateral cervical spine are dissected effectively and usually without difficulty. With the patient in a supine position, the skin incision can be horizontal or longitudinal, parallel to the course of the sternocleidomastoid muscle. With this approach, the surgeon has direct access to the vertebral bodies and the intervertebral discs, so that discectomy, removal of osteophytes, and, where required, corpectomy of the vertebrae are easy and feasible [1-3].

However, anterior spinal surgery carries a number of risks and potential complications. Implant failure and graft migration, which usually occur anteriorly, may result in partial kyphosis, dysphagia, airway obstruction, esophagus perforation, or pressure on the carotid artery leading to neurological symptoms. Also, in cases where an autologous bone graft is used, increased donor-site morbidity and the development of nonunion have been recorded [4-5]. Employing a poor surgical technique during the removal of osteophytes may lead to the injury of dura matter and cerebrospinal fluid leakage [6].

The process of swallowing is a vital but possibly underestimated function that involves emptying the nasopharynx and oropharynx and subsequently coordinating to close the nasopharynx and larynx to prevent aspiration [7]. The process of normal swallowing involves the fine collaboration of more than 25 pairs of muscles in the oral cavity, pharynx, larynx, and esophagus. There are three phases in the swallowing process: oral, pharyngeal, and esophageal. Dysphagia, which is defined as a dysfunction of normal swallowing, can occur during any or all of the three phases of swallowing [8-9].

There are three main types of dysphagia: acute, chronic, and progressive. Acute dysphagia mainly occurs after cervical head surgeries, neurosurgery, injuries, craniocerebral injuries, and vascular strokes. However, in genetic syndromes, such as cerebral palsy and developmental disorders, dysphagia is usually chronic. Progressive dysphagia is commonly found in neurological degenerative diseases. Symptoms of dysphagia include coughing or choking, foreign body sensation in the throat, food remainings in the mouth after swallowing, uncoordinated laryngeal movement, weight loss, and unexplained, recurrent nausea and/or vomiting. Dysphagia can increase the duration of hospitalization, affect recovery, and also endanger the quality of life of patients with spinal cord injury (SCI) in terms of oral nutrition and the ability to communicate. Hence, any difficulty in receiving, managing, promoting, and swallowing saliva, food, fluids, and substances of any composition, even medicines in general, entails a number of complex problems that characterize people with dysphagia [10-12].

While dysphagia after the anterior spinal cervical approach is common, it is fortunately transient in most cases [13-14]. It may be caused by prevertebral soft tissue swelling, hematoma, bleeding, nerve injury, or inflammation associated with anterior cervical hardware irritation or esophageal retraction [14]. It occurs more frequently in patients who have undergone spinal fusion of more than one level and does not depend on anterior plate placement [6]. Older patients (aged >60 years) and those with pre-existing dysphagia (on the grounds of myelopathy) are at high risk for postoperative dysphagia [15]. Despite its high incidence, prolonged postoperative dysphagia is poorly understood; its pathogenesis remains relatively unknown, and its risk factors are still widely debated. The aim of this study is to review the incidence, pathogenesis, diagnosis, and modes of prevention of dysphagia in ACDF patients.

A literature review was conducted based on the PubMed internet database, following the PRISMA guidelines, with the use of the EndNote X3 software (Thompson Reuters) [16]. Article titles were searched with the use of the following keywords: “dysphagia” and “anterior cervical diskectomy and fusion” or “ACDF”. The search was limited to prospective clinical studies evaluating dysphagia after ACDF surgery. Studies published in non-English languages, retrospective studies, cadaveric studies, reviews, case reports, study protocols, and commentary studies were excluded.

## Review

Results

**Figure 1:**
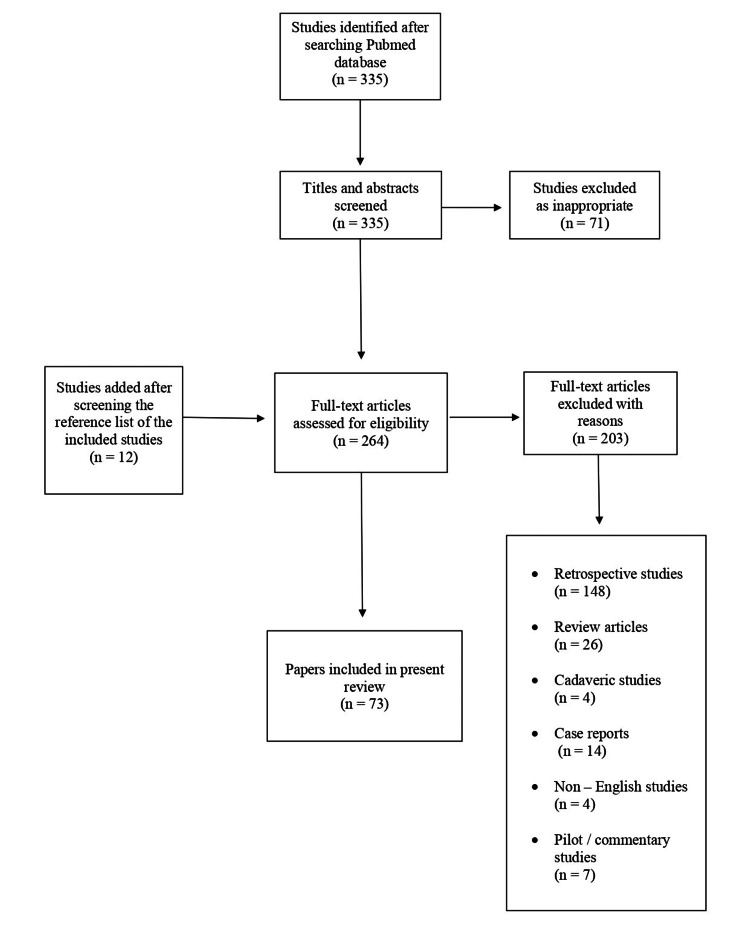
Study selection flowchart

Initially, 335 studies were identified after a primary search on the PubMed electronic database. After the screening of titles and abstracts, 71 articles were excluded. Among the remaining 264 studies, 203 were rejected for creating a more homogenous patient cohort sample, as 148 were identified as retrospective studies, 26 were review articles, 14 were case report presentations, seven were pilot/commentary studies, four were cadaveric studies, and four papers were not in English. After a full-text analysis of the remaining studies, 12 additional studies that were found in the reference list of the already included studies were added, leaving 73 studies for the final analysis (Figure 1).

Incidence of Dysphagia and ACDF

The incidence of postoperative dysphagia after ACDF has been reported in a plethora of studies, with varying results (Table 1) [1-95]. Dysphagia is considered to be a common complication in patients after ACDF, as in 50% of cases it was found video-radiographically after surgery. Although dysphagia may persist for months or years, the symptoms are typically transient. Thus, the timing of evaluation and follow-up influences the rate of diagnosis. Various studies have estimated the rate of dysphagia to range from 1 to 79% [17-21].

**Table 1:** Dysphagia incidence and ACDF: study characteristics ACDF: anterior cervical discectomy and fusion; ACCF: anterior cervical corpectomy and fusion

Authors, year	Study design	Patient details	Interventions (operated levels)	Follow-up time intervals	Graft type
Riley et al., 2005 [43]	Registry cohort study	N=454, mean age: 48.2 years, % male: 52.2	ACDF	3, 6, and 24 months	N/A
Bazaz et al., 2002 [19]	Prospective cohort	N=249, mean age: 52 years, no. of males: 52	ACDF or ACCF with or without plate or graft revision	6 months	Autograft
Haller et al., 2022 [21]	Prospective study	N=56, mean age: 60 years, no. of females: 33	ACDF	2, 12, and 24 months	N/A
Kepler et al., 2012 [79]	Prospective cohort	N=43	ACDF one level: n=15, two levels: n=28	1.5 months	Autograft or allograft
Bruneau et al., 2001 [22]	Prospective cohort	N=54	ACDF one level: n=40, two levels: n=14	24.6 months	Hydroxyapatite
Lied et al., 2013 [23]	Prospective single-center	N=96, mean age: 49 years, no. of females: 33	ACDF one level: n=60, two levels: n=36	6 months	N/A
Opsenak et al., 2019 [31]	Prospective single-center study	N=73	ACDF (zero-profile)	6 weeks; 3, 6, and 12 months	Allograft
Srikhande et al., 2019 [24]	Prospective study	N=100, mean age: 47.2 years, no. of females: 14	ACDF one level: n ¼ 95, two levels: n ¼ 5	24 months	Autograft
Frempong-Boadu et al., 2002 [20]	Prospective cohort	N=23, mean age: 59 years, % male: 95.6	ACDF (up to three levels)	1 week post-op, 12 months	Allograft
Mendoza-Lattes et al., 2008 [36]	Prospective cohort	N=17, mean age: 47.8 years, % male: 35.3	ACDF with either dynamic retraction or static retraction one level: n=7, two levels: n=10	1st day post-op, 6 weeks, 3 and 6 months	Allograft
Papavero et al., 2007 [35]	Prospective cohort	N=92, mean age: NR, % male: 57.6	ACDF using predominantly a left-sided Smith-Robinson approach	1st, 3rd, and 5th post-op day	N/A
Hou et al., 2014 [93]	Prospective cohort	N=196	ACDF one level: n=108, two levels: n=88	22.5 months	Autograft
Song and Lee, 2006 [94]	Prospective cohort	N=39, mean age: 46.3 years	ACDF one level	24 months	Autograft
Bolesta et al., 2000 [95]	Prospective cohort	N=15, mean age: 51 years, no. of males: 5, no. of females: 10	ACDF three levels: n=12, four levels: n=3	42 months	Autograft
O'Donohoe et al., 2020 [51]	Prospective cohort	N=25, mean age: 55.79 years, no. of females: 17	ACDF one level	2 years	Allograft
Lee et al., 2005 [52]	Prospective cohort	N=156	ACDF with 2 different plates: Atlantis and Zephir	1, 2, 6, 12, and 24 months	Allograft
Grasso et al., 2018 [58]	Prospective study	N=100	ACDF (zero-profile implants)	4 years	N/A
Zhang et al., 2016 [56]	Prospective cohort	N=50, mean age: 50.65 years, no. of males: 24, no. of females: 26	ACDF (zero-profile implants and anterior plate and cage)	2 years	N/A
De Leo-Vargas et al., 2019 [57]	Prospective study	N=53, mean age: 58.8 years	ACDF	6.7 months	N/A
Scholz et al., 2011 [62]	Prospective study	N=38, mean age: 53.7 years, no. of males: 24, no. of females: 14	ACDF (zero-profile implants)	6 months	N/A
El Baz et al., 2019 [63]	Prospective study	N=25, no. of males: 21, no. of females: 4	ACDF (zero-profile cage)	6 months (average follow-up time)	N/A
Gerszten et al., 2016 [64]	Prospective study	N=68, mean age: 56 years, no. of males: 51, no of females: 17	ACDF (zero-profile fixation and stand-alone PEEK cages)	6 months	Allograft
Grasso et al., 2014 [65]	Prospective study	N=32, mean age: 59.8 years, no. of males: 18, no of females: 14	ACDF (ROI-C cages)	6 weeks; 3, 6, 12, and 24 months	Allograft (cadaveric bony tissue)
Chen et al., 2015 [66]	Prospective study	N=69, mean age: 49.2 years, no. of males: 41, no of females: 28	ACDF (Zero-P spacer, two-level fusion)	2-6 months	N/A
He et al., 2018 [67]	Prospective randomized trial	N=104, mean age: 57.4 years, no. of males: 55, no of females: 49	ACDF (zero-profile implants and traditional anterior cervical plate)	24 months	Autograft and allograft
Qizhi et al., 2016 [68]	Prospective cohort	N=17, mean age: 60.7 years, no. of males: 12, no. of females: 5	ACDF (zero-profile implants)	48, 59 months	N/A

Dysphagia Rate and Evaluation

**Figure 2:**
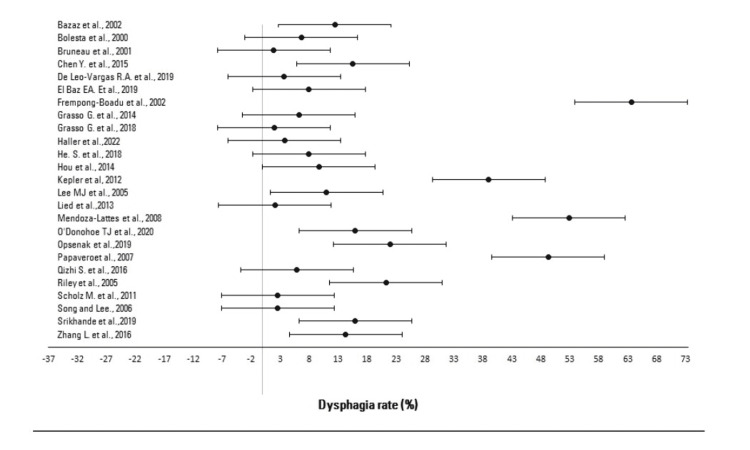
Dysphagia rates and 95% confidence intervals (CIs) as per studies correlating dysphagia with ACDF, expressed in a forest plot for meta-analysis ACDF: anterior cervical discectomy and fusion

A mean dysphagia rate of 19.4% (95% CI: 9.6%-29.1%) based on the included studies correlating dysphagia directly with ACDF procedures was calculated (Table 2). The upper and lower value intervals expressed as a percentage of each reported dysphagia rate of the included studies were calculated based on the 95% confidence interval, as presented in Table 2 and Figure 2. A crucial evaluation point is that across the combined interstudy population, studies reported dysphagia at different time intervals during the follow-up period, and hence the follow-up time holds no homogeneity, being a variable factor impacting the data analysis. Therefore, the dysphagia rates reported at the latest follow-up time in the studies included were chosen to be evaluated. Because of this limitation and the concomitant lack of studies with a consistent control follow-up interval time, further research is recommended to be performed on this topic. Moreover, the variation of the reported incidence may be attributed to the lack of standard criteria for its diagnosis, measurement, and the retrospective nature of many published studies. Retrospective studies would more often underreport postoperative complications [25]; moreover, the included studies used different dysphagia classification schemes, defining dysphagia differently or not at all, as discussed later.

**Table 2:** Dysphagia rates and 95% confidence intervals (CIs) as per the studies

Study	Dysphagia rate (95% CI)
Bazaz et al., 2002 [19]	12.5% (2.8%-22.2%)
Bolesta et al., 2000 [95]	6.7% (-3.0%-16.4%)
Bruneau et al., 2001 [22]	1.9% (-7.8%-11.6%)
Chen et al., 2015 [66]	15.6% (5.9%-25.3%)
De Leo-Vargas et al., 2019 [57]	3.7% (-6.0%-13.4%)
El Baz et al., 2019 [63]	8.0% (-1.7%-17.7%)
Frempong-Boadu et al., 2002 [20]	63.6% (53.9%-73.3%)
Grasso et al., 2014 [65]	6.25% (-3.5%-15.9%)
Grasso et al., 2018 [58]	2.0% (-7.7%-11.7%)
Haller et al., 2022 [21]	3.8% (-5.9%-13.5%)
He et al., 2018 [67]	8.0% (-1.7%-17.7%)
Hou et al., 2014 [93]	9.7% (-0.02%-19.4%)
Kepler et al., 2012 [79]	39.0% (29.3%-48.7%)
Lee et al., 2005 [52]	11.0% (1.3%-20.7%)
Lied et al., 2013 [23]	2.1% (-7.6%-11.8%)
Mendoza-Lattes et al., 2008 [36]	52.9% (43.2%-62.6%)
O'Donohoe et al., 2020 [51]	16.0% (6.3%-25.7%)
Opsenak et al., 2019 [31]	22.0% (12.3%-31.7%)
Papavero et al., 2007 [35]	49.3% (39.6%-59.0%)
Qizhi et al., 2016 [68]	5.9% (-3.8%-15.6%)
Riley et al., 2005 [43]	21.3% (11.6%-31.0%)
Scholz et al., 2011 [62]	2.6% (-7.1%-12.3%)
Song and Lee, 2006 [94]	2.6% (-7.1%-12.3%)
Srikhande et al., 2019 [24]	16.0% (6.3%-25.7%)
Zhang et al., 2016 [56]	14.3% (4.6%-24.0%)
Mean dysphagia rate	19.4% (9.6%-29.1%)

Risk Factors

Dysphagia as a complication after ACDF procedures has a multivariable characteristic. Many factors play a crucial role in leading to dysphagia and thereby affecting the dysphagia rates reported in the studies, some of which are patient sex, the surgical approach, the different ACDF devices utilized, the graft type, rhBMP-2 use, and the administration of steroids. By reviewing the current literature on the topic, an attempt was made to identify, compare, and evaluate those factors individually and the effect they might have in terms of leading to dysphagia.

Other causes, such as movement of structures, pharyngeal edema, or paralysis of the vocal cords, are also known to have an effect on postoperative dysphagia. Individuals who undergo surgery for cervical spine disease with anterior or posterior access tend to develop oropharyngeal dysphagia, which is often severe during the first month and progressively diminishes in intensity over time [26]. Multivariable analyses have identified smoking status, prior cervical surgery, preoperative C2-C7 angle, preoperative dysphagia, preoperative chronic obstructive pulmonary disease, multilevel surgery, and intraoperative steroid use as significant risk factors for dysphagia after ACDF [26-30]. However, the prospective study by Opsenak et al. failed to confirm these findings [31]. Prevertebral soft-tissue swelling of more than 5 mm and change of cervical alignment of more than 5 degrees are known predictors of postoperative dysphagia after ACDF [20,30,32]. Prevertebral soft-tissue swelling after ACDF resolves and returns to presurgery status within one to three months in the pharyngeal airway (C3) and within three to six months in the laryngeal airway (C6).

Female gender and obesity have been determined to have an influence on prevertebral soft tissue swelling after ACDF [26,33]. Operation time has been correlated with the severity of postoperative dysphagia [17]. Psychiatric disorders and preoperative opioid use are known predisposing factors of postoperative chronic dysphagia following ACDF [34]. No correlation between intraoperative esophagus retraction and postoperative dysphagia has been documented [36]. On the contrary, Mendoza-Lattes et al. have observed that during ACDF, patients were exposed to higher intraoperative esophageal pressure and decreased esophageal mucosal blood flow during surgical retraction. Dynamic retraction seemed to be associated with a lower prevalence of postoperative dysphagia [36].

Surgical Approach

The anterior approach of ACDF is highly associated with the development of dysphagia. Patients who underwent anterior or combined approach had a disproportionately high incidence of swallowing disorders. A randomized controlled trial compared the rate of postoperative dysphagia among patients who underwent two different approaches for ACDF. The authors concluded that, for the better prevention of dysphagia, an anterior approach lateral to the omohyoid muscled should be selected if the level of surgery involves C3-C4. For C6-C7 surgery, however, a left-sided anterior approach medial to the omohyoid muscle should be used [37]. Of note, 85% of patients with dysphagia after cervical spine surgery have been subjected to ACDF [38]. The placement of anterior cervical plates results in increased intraesophageal pressure when it is done in the vertebra C5-C6. This is true regardless of whether the placement of the plates occurs from the C3 to the C6 vertebra or individually to the C5-C6. In addition, cervical disc placement appears to require less esophageal friction and reduces esophageal pressure when compared to anterior plaque placement [39]. The reported rates of postoperative dysphagia are significantly higher in ACDF in comparison with posterior decompression and fusion [40-42]. Multiple cervical levels of ACDF represent a significantly higher postoperative risk for swallowing dysfunction, as compared with one-level ACDF [18,20,43]. Four-level ACDF has been associated with a 32% rate of transient dysphagia [43].

ACDF Devices and Disc Replacement

The comparison between ACDF and anterior disk replacement in terms of postoperative dysphagia has shown conflicting results. Anderson et al. reported that the incidence of postoperative dysphagia is higher in anterior disk replacement surgery in comparison to ACDF [44]. Other studies have found no significant difference in rates of postoperative dysphagia between ACDF and cervical disk arthroplasty [45]. Philips et al. found a statistically lower rate of prolonged dysphagia in patients subjected to cervical disk replacement [46]. Shi et al. found no significant difference in postoperative swallowing dysfunction when ACDF with zero-profile cages was compared to cervical disc arthroplasty (CDA) with Discover prosthesis [47]. Bryan(®) CDA has been found to have a significantly reduced rate of dysphagia compared to ACDF [48]. In a prospective randomized clinical study by McAfee et al., the incidence of postoperative dysphagia and the long-term resolution of the dysphagia were found to be greatly improved in the disk arthroplasty group in comparison to the instrumented ACDF control group [49].

The impact of implants used for ACDF has been extensively studied. Modular plates have been associated with a trend toward lower dysphagia in comparison to traditional plates [50]. The placement of larger plates may prolong retraction time but is not associated with higher rates of dysphagia, according to a prospective study by O’ Donohoe et al. [51]. Smaller and smoother plates may reduce the incidence of postoperative dysphagia [52]. Plate thickness or preoperative osteophyte height does not seem to affect the risk of postoperative dysphagia after ACDF. Plates at C3 have a trend for an increased risk for postoperative dysphagia [53].

Zero-profile devices for ACDF have been widely associated with lower rates of postoperative dysphagia, compared to conventional implants [54-56]. The incidence of postoperative dysphagia in patients undergoing ACDF with zero-profile stand-alone cervical cages has been reported as 3.7% [57]. ACDF with the use of zero-profile intervertebral cages has shown a rate of transient postoperative dysphagia at 1.1%-7.9% [58-62]. Among 25 patients treated with ACDF with a zero-profile cage, three patients presented with mild transient dysphagia that resolved at two weeks and two patients had moderate dysphagia that resolved at five weeks [63]. In a prospective cohort study, the rate of dysphagia after ACDF with zero-profile devices that included titanium screw fixation was 12%, while the corresponding rate was 9% when PEEK cages were used [64]. When ROI-C cages were used for ACDF, the reported rate of transient postoperative dysphagia was 3.1% [65]. Two-level ACDF with the zero-profile spacer has a higher incidence of postoperative dysphagia compared with ACDF with plates and screws [66]. In multilevel ACDF, He et al. found a 0% rate of dysphagia when zero-profile constructs were used, in comparison to an 8% rate when traditional plate and screws were used [67]. Qizhi et al. reported a 5.9% rate of dysphagia after ACDF with zero-profile devices [68]. ACDF with intervertebral cages may have a higher rate of postoperative dysphagia in comparison to anterior instrumentation with plates and screws, according to Zavras et al. [69].

Graft Type and rhBMP-2

The use of grafts or growth factors may affect the development of dysphagia. Studies that performed ACDF with autograft fusion reported an overall dysphagia rate of 9.9%, whereas the use of allograft fusion resulted in a rate of 7.9%. The highest rate of dysphagia (20.2%) among studies using autograft fusion was reported by Bazaz et al. [19]. Among 72 ACDF patients with the use of bioabsorbable cervical spacer treated with low-dose rhBMP-2, only two patients suffered from excessive postoperative dysphagia [70]. The addition of rhBMP-2 to ACDF has been associated with increased postoperative dysphagia seven days after surgery [71-72]. Randomized controlled studies have shown that locally administered steroids on a collagen sponge significantly reduce the incidence and severity of postoperative dysphagia following ACDF using low-dose rhBMP-2 [73]. ACDF with intervertebral cages and the addition of BMP-7 has been reported to have a 1.6% rate of postoperative dysphagia [74]. ACDF with PEEK cages and the addition of rhBMP-2 placed on a type I collagen sponge and titanium plates was accompanied by up to 47% rate of swallowing problems two weeks after surgery. Swallowing scores dramatically improved six months after ACDF [75]. ACDF with titanium cages or anterior locking plating and tricortical iliac crest graft was associated with a 15% rate of dysphagia according to a prospective comparative study by Singh et al. [76].

Diagnosing Dysphagia

The Eating Assessment Tool (EAT-10) and the Bazaz Dysphagia score are two widely used clinical scoring systems for the measurement of dysphagia in clinical studies [77,78]. The Bazaz score classifies dysphagia into none, mild, moderate, or severe based on its frequency and the kinds of foods that precipitate dysphagia [19]. In lateral cervical X-rays, there is a significant increase in anterior cervical soft-tissue swelling after ACDF. However, the width of prevertebral soft tissue is not associated with postoperative dysphagia [79]. According to Song et al., the measurement of prevertebral soft-tissue swelling with the use of consecutive cervical lateral radiographs after ACDF may contribute to the diagnosis of postoperative dysphagia [34].

As swallowing is a dynamic process, the videofluoroscopic swallow study (VFSS) is probably the most appropriate method of evaluating this normal function. Its aim is to detect and evaluate the physiology of all stages of swallowing, the reason why the examinee has a problem, and also to guide the therapist in choosing appropriate strategies by which the patient's nutrition will be safe and adequate for survival. The patient is asked to manage foods of any composition impregnated with barium, while at the same time the whole procedure is videotaped. VFSS studies have shown that patients after ACDF with their highest surgery level at C3 and C4 had more severe swallowing dysfunction and significantly increased soft-tissue thickness [80]. For patients with persisting dysphagia, VFSS has shown significant impairments in pharyngeal constriction, hyoid displacement, and pharyngoesophageal segment opening [40]. VFSS studies have reported that the incidences of dysphagia measured by the Bazaz Dysphagia score were 83.0% at one week and 59.6% at one month after ACDF [81].

Fiberoptic endoscopic evaluation of swallowing (FEES) is an examination performed by a doctor by using a flexible endoscope and a screen that is available to monitor the examination. This examination allows the control of anatomical structures (nasopharynx and larynx) involved in swallowing and phonation, the delay or absence of the pharyngeal or laryngeal reflex, the correct direction and timing of the bolus, the control of its early escape into the pharynx, the reduced sensation, the presence of residues in the lingual epiglottis or in the opioid pits, and the aspiration or the penetration [31]. The rate of dysphagia after ACDF as assessed by FEES has been reported to be 37% [82].

Prevention

Steroids have been administered intraoperatively with the aim to reduce dysphagia risk during ACDF; however, the results have been contradictory. Prospective randomized trials have not demonstrated any benefit of local or intravenous intraoperative administration of steroids in terms of patient-reported swallowing function or prevertebral soft-tissue swelling following ACDF [83-84]. Intraoperative administration of local anesthetics to the retropharyngeal space did not reduce dysphagia symptoms after ACDF [85]. Other prospective studies have observed that the local administration of steroids after multilevel ACDF can decrease postoperative soft-tissue swelling and the rate of postoperative dysphagia [86-87]. Retropharyngeal administration of steroids for the prevention of postoperative dysphagia is associated with a decreased rate of radiographic fusion or delayed fusion in ACDF surgery [88-89]. Jenkins et al. have observed that both local and intravenous intraoperative administration of steroids is associated with decreased dysphagia rates, when compared to the control group, after ACDF (p=0.014) [90]. Similarly, Jeyamohan et al. found that perioperative administration of dexamethasone significantly improved swallowing function and airway edema, but did not affect long-term swallowing status [89]. Intraoperative placement of an esophageal temperature probe significantly improved postoperative dysphagia scores in patients undergoing two-level ACDF at two weeks and six months postoperatively [91]. The adjustment of endotracheal tube-cuff pressure in ACDF does not have a significant impact on the incidence of postoperative dysphagia [92].

## Conclusions

Dysphagia is an established multifactorial complication of ACDF with varying incidence rates. This literature review focused on identifying the rate of dysphagia and the various risk factors leading to this complication by comparing and evaluating the current literature with a wide spectrum of heterogeneity concerning patients, surgeons, and surgical techniques. A mean dysphagia rate of 19.4% (95% CI: 9.6%-29.1%) based on the findings of included studies correlating dysphagia directly with ACDF procedures was calculated. Various established risk factors leading to dysphagia include the female sex, smoking, the surgical approach, rhBMP-2 use, and multilevel surgery, while zero-profile devices seem to reduce dysphagia risk. The diagnosis is based on clinical and radiological findings, especially prevertebral soft-tissue swelling. However, videofluoroscopic and endoscopic studies have been recently used for the evaluation of dysphagia. The role of the local administration of steroids in the prevention of dysphagia has not yet been clarified. This review underscores the existing rudimentary understanding of the problem of dysphagia after ACDF procedures and highlights the need for more sensitive, factor-specific studies for gaining deeper insights into the impact of various risk factors on the rate of dysphagia.
